# Engineered G protein coupled receptors reveal independent regulation of internalization, desensitization and acute signaling

**DOI:** 10.1186/1741-7007-3-3

**Published:** 2005-02-11

**Authors:** Kimberly Scearce-Levie, Michael D Lieberman, Heather H Elliott, Bruce R Conklin

**Affiliations:** 1 The Gladstone Institute of Neurological Disease and the Gladstone Institute of Cardiovascular Disease, San Francisco CA 94158 USA; 2 Departments of Medicine and Molecular and Cellular Pharmacology, University of California, San Francisco, CA, 94143 USA

## Abstract

**Background:**

The physiological regulation of G protein-coupled receptors, through desensitization and internalization, modulates the length of the receptor signal and may influence the development of tolerance and dependence in response to chronic drug treatment. To explore the importance of receptor regulation, we engineered a series of G_i_-coupled receptors that differ in signal length, degree of agonist-induced internalization, and ability to induce adenylyl cyclase superactivation. All of these receptors, based on the kappa opioid receptor, were modified to be receptors activated solely by synthetic ligands (RASSLs). This modification allows us to compare receptors that have the same ligands and effectors, but differ only in desensitization and internalization.

**Results:**

Removal of phosphorylation sites in the C-terminus of the RASSL resulted in a mutant that was resistant to internalization and less prone to desensitization. Replacement of the C-terminus of the RASSL with the corresponding portion of the mu opioid receptor eliminated the induction of AC superactivation, without disrupting agonist-induced desensitization or internalization. Surprisingly, removal of phosphorylation sites from this chimera resulted in a receptor that is constitutively internalized, even in the absence of agonist. However, the receptor still signals and desensitizes in response to agonist, indicating normal G-protein coupling and partial membrane expression.

**Conclusions:**

These studies reveal that internalization, desensitization and adenylyl cyclase superactivation, all processes that decrease chronic G_i_-receptor signals, are independently regulated. Furthermore, specific mutations can radically alter superactivation or internalization without affecting the efficacy of acute G_i _signaling. These mutant RASSLs will be useful for further elucidating the temporal dynamics of the signaling of G protein-coupled receptors *in vitro *and *in vivo*.

## Background

The specificity, diversity, and physiological importance of G protein-coupled receptors (GPCR) have made these receptors excellent drug targets. It is becoming clear that the regulation of the GPCR itself – its location, stability, and signal duration – is a key component of the signaling process [1,2] The length of a GPCR signal can be modulated by receptor desensitization (decrease in receptor responsiveness) and receptor internalization (trafficking of receptors to endocytotic vesicles). The cell can also respond to prolonged activation by upregulating compensatory pathways. For example, prolonged signaling through a G_i_-coupled receptor inhibits adenylyl cyclase (AC), while paradoxically increasing the ability of the G_s_-coupled pathway to stimulate AC, a phenomenon known as AC superactivation [3]. Such regulatory mechanisms may contribute to the development of drug tolerance and dependence, including the response to chronic opiate use [4].

The complex effects of drugs at multiple receptor subtypes in multiple tissues have made it difficult to isolate the relative contributions of GPCR regulation, ligand binding, effector coupling, drug metabolism, and cellular downregulation machinery. Even if two receptors couple to the same signaling pathway, the physiological effects of their activation could vary tremendously depending on the pharmacokinetics of the ligands, the cell type expressing the receptors, and the interactions with desensitization mechanisms. An engineered family of receptors that share the same ligand binding and effector coupling, yet have discrete mutations that cause them to internalize or desensitize differentially, would help pinpoint the physiological consequences of GPCR desensitization. This is especially important in the light of recent evidence showing dramatically different endocytosis and signaling profiles of mu opioid receptors (MOR) in response to different ligands [5,6]. In addition, understanding the signals that allow transmembrane proteins to be more or less resistant to endocytosis will improve our understanding of endocytosis as a general regulatory mechanism, as it has been implicated in the regulation of signaling of growth factor receptors [7,8] and ion channels [9-11].

Our laboratory has engineered a G_i_-coupled receptor that is insensitive to endogenous ligands but can still respond to the synthetic small-molecule agonist spiradoline [12,13]. This receptor activated solely by a synthetic ligand (RASSL) was based on the kappa opioid receptor (KOR). In the original RASSLs, exchanging the second extracellular loop of the KOR with the corresponding sequence from the delta opioid receptor, and making an additional point mutation (Q297E), resulted in a receptor with 1/2,000 of the response to dynorphin and other endogenous peptides relative to the wild-type KOR. However, the response of this RASSL to spiradoline was not altered. RASSLs can be expressed in a tissue-specific manner in transgenic mice, allowing direct control of G_i_-mediated physiological responses such as heart rate [14]. It has also been recently used to help identify the mammalian sweet receptor by expressing it in mouse taste buds [15]. To investigate the endocytosis and desensitization of GPCRs, we have since developed four new RASSLs. To visualize these RASSLs in living cells, we fused the green fluorescent protein (GFP) to the N-terminus (outer portion) of the RASSL resulting in Rog (RASSL opioid green). Given the well-documented role of the C-terminal region in the desensitization and internalization of GPCRs [1,16-18], we made a series of C-terminal mutant RASSLs designed to desensitize and internalize at different rates.

This novel receptor system offers an opportunity to test specific hypotheses about the relationship between receptor sequence and receptor regulation, without requiring the use of multiple ligands that might have different effects on the signal and the regulation of receptors. Because RASSLs lack endogenous agonists, they allow greater control of the timing and specificity of activation than is possible with endogenous receptors. In these studies, we test how the removal of phosphorylation sites from the C-terminal regions of a G_i_-coupled RASSL alters the receptor's internalization, desensitization, and induction of AC superactivation. Since it is well established that the endogenous mu and kappa opioid receptors differ in these properties, we also explore the regulation of kappa opioid RASSLs bearing specific portions of the mu opioid receptor C-terminal sequence. The cell culture experiments presented here provide a basis for *in vivo *studies in complex tissues such as the nervous system. Insight gained through these experiments may help explain the differences seen *in vivo *between different endogenous G_i_-coupled receptors, improving our understanding of the contribution of receptor regulation to the physiological response to agonists and our overall understanding of GPCR signal regulation.

## Results

### Rog, a GFP-tagged RASSL, signals appropriately

Although an N-terminal GFP tag does not interfere with the function of the human KOR [19], we wanted to confirm that this tag does not modify the signaling properties of Rog, a KOR-based RASSL. Rog was transiently transfected into CHO cells along with a chimeric G_qi5 _protein [20] that couples to G_i_-coupled receptors but signals through the G_q _pathway and therefore stimulates calcium release. Using this transient calcium response as a measure of G_i _activation, we showed by FLIPR assay that Rog responded dose-dependently to spiradoline, but not to a range of doses of dynorphin, the endogenous ligand that activates the wild-type KOR (Figure 2A). Therefore, Rog, like its predecessors Ro1 and Ro2 [12], meets the criteria for a RASSL.

We evaluated all of the engineered receptors for their ability to affect cAMP formation. G_i _signaling decreases intracellular cAMP levels by directly inhibiting adenylyl cyclase. Activation of these G_i_-coupled receptors with spiradoline was therefore expected to inhibit forskolin-induced cAMP accumulation in a dose-dependent manner. Indeed, under basal conditions, 15-min treatment with spiradoline activated Rog, Rog-A, Rog-μ and Rog-μA, as indicated by inhibition of forskolin-induced accumulation of cAMP (Figure 5, Table 1). Despite differences in C-terminal amino acid sequence, the RASSLs showed no significant differences in their ability to inhibit cAMP accumulation after acute activation with spiradoline (Table 1). EC50 and cAMP inhibition values for all of the RASSLs were similar to those seen with the human KOR in the same assay. In a representative experiment, the EC50 for KOR was 0.91 nM spiradoline and cAMP inhibition was 68.6%.

### Rog is internalized by agonist treatment

The GFP tag on Rog allows direct observation of agonist-induced receptor internalization by confocal microscopy (Figure 2B). In untreated cells, the receptor was visible primarily on the plasma membrane. One hour after spiradoline treatment (10–100 μM), the receptor was observed in bright, punctate intracellular vesicles. Dynorphin (100 μM), in contrast, did not lead to significant internalization of the receptor (Figure 2B). An ELISA that detects only cell-surface receptors was used to quantify the extent of receptor internalization after spiradoline treatment. With increasing doses of spiradoline, fewer receptors were detected on the cell surface, culminating in an approximately 45% loss of cell-surface receptors at the maximal dose of 100 μM (Figure 2C). The same dose of spiradoline resulted in a similar 47% loss of KOR from the cell surface (not shown). The time course of internalization in response to 1 μM spiradoline was relatively rapid, with significant receptor loss apparent within 5 min (Figure 2C). Maximal receptor loss was detected approximately 20 min after agonist treatment began.

### Rog-A is resistant to agonist-induced internalization

**Figure 1:**
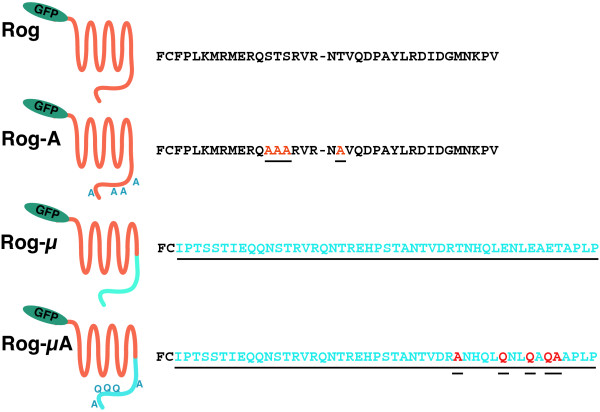
**Design of RASSL variants. **The design of each RASSL variant is shown on the left. Differences in amino acid sequences of the receptor variants are shown in the C-terminal alignments, right. Sequence derived from the MOR are dark, while KOR sequences are light. All mutated residues are underlined.

To determine the role of C-terminal phosphorylation sites in receptor regulation, we examined spiradoline-induced internalization of Rog-A, a mutated version of Rog in which four C-terminal phosphorylation sites were mutated to alanine (Figure 1). HEK293 cells stably transfected with Rog-A were treated with 10 μM spiradoline, a dose sufficient to cause internalization of most Rog receptors (Figure 3A, left). One hour after spiradoline treatment, most Rog-A receptors appeared to remain in the membrane (Figure 3A, center). Quantification by cell-surface ELISA showed significantly less loss of cell-surface receptors for Rog-A than for Rog at spiradoline doses of 0.1–100 μM (Figure 3B). ANOVA indicated a main effect of drug dose (F_10,20 _= 66.53, p < 0.0001) and a main effect of receptor type (F_1,20 _= 55.29, p < 0.0001). As observed with Rog, maximal internalization of Rog-A in response to 1 μM spiradoline occurred after 20 min of drug treatment (Figure 3B). However, in contrast to Rog, fewer than 10% of the Rog-A receptors were internalized at that time point. ANOVA of the time course data indicated a main effect of length of treatment (F_11,47 _= 11.39, p < 0.0001), a main effect of receptor type (F_1,47 _= 203.16, p < 0.0001), and an interaction between receptor type and treatment length (F_11,47 _= 2.27, p < 0.02). These results suggest that C-terminal phosphorylation promotes receptor internalization. Activation of Rog-A inhibits cAMP as fully as Rog (Table 1), indicating that the reduced internalization does not alter acute signaling.

### Rog-μ more readily internalizes in response to spiradoline

Since the mu opioid receptor (MOR) internalizes more readily than the KOR, we made Rog-μ, a chimeric receptor in which the entire intracellular portion of the C-terminus was replaced with the corresponding MOR sequence (Figure 1). Rog-μ was expected to internalize to a greater extent than Rog in response to spiradoline. Confocal microscopy showed nearly complete internalization of Rog-μ after one hour of treatment with 10 μM spiradoline (Figure 3A, right). A cell-surface ELISA revealed 25–30% internalization of Rog-μ at low doses of spiradoline, ranging from 0.01 to 0.1 μM (Figure 3C). Little internalization of Rog or Rog-A has been observed at these doses (Figures 3B and 3C). At higher doses of spiradoline, no difference in internalization between Rog and Rog-μ was observed. ANOVA indicated a main effect of drug dose (F_9,52 _= 55.79, p < 0.0001) and an interaction between receptor type and drug dose (F_9,52 _= 3.89, p < 0.0008). Post-hoc Scheffé analysis shows significant differences between Rog and Rog-μ at 0.01 μM (p = .024) and 0.1 μM (p = .016) doses of spiradoline. When cells were treated with 1 μM spiradoline for differing lengths of time, there was no detectable difference in the time course of internalization of Rog and Rog-μ (Figure 3C). ANOVA indicated a main effect of time (F_11,48 _= 53.20, p < 0.0001), with no effect of receptor type. There was also no difference in cAMP inhibition after spiradoline activation of Rog and Rog-μ (Table 1).

### Constitutive internalization of Rog-μA

A variant of Rog-μ, known as Rog-μA, also has MOR sequence at the C-terminus, but five serine and glutamic acid residues at the C-terminus were mutated (Figure 1). These mutations were predicted to render Rog-μA more resistant to internalization than Rog-μ [18]. However, in several independent stably and transiently transfected cell lines (HEK293 and rat1a), Rog-μA always had significantly lower cell-surface expression than other receptors. As shown by cell-surface ELISA of one representative group of stably transfected HEK293 lines, Rog-μA was expressed at 28% of the level of Rog, and 41% of Rog-μ (Figure 4A). Despite the low cell-surface expression, Rog-μA signals as well as the other RASSLs after acute spiradoline treatment (Table 1).

**Figure 2:**
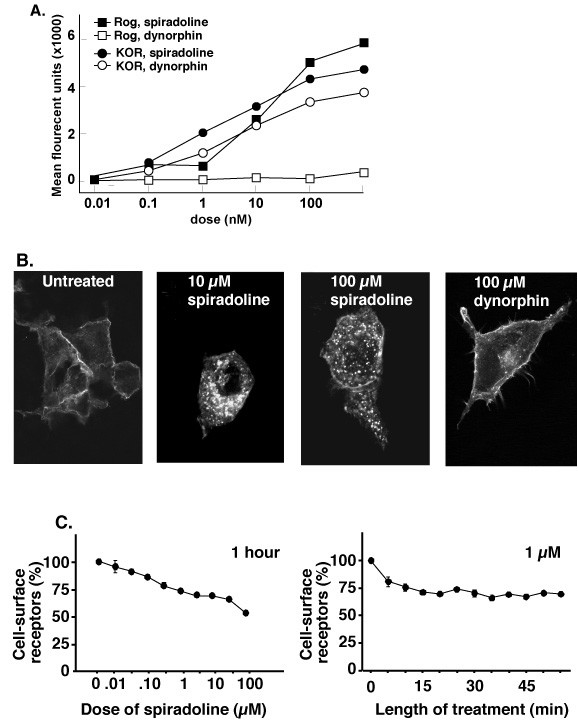
**Agonist-induced signaling and internalization of Rog. **(A) Maximum calcium response plotted as a function of spiradoline dose for cells transfected with Rog or the wild-type KOR and treated with dynorphin or spiradoline. (B) Confocal micrographs show representative internalization of GFP-tagged Rog receptors 1 h after treatment with 10 μM or 100 μM spiradoline. Dynorphin at 100 μM (far right) caused little internalization in this assay. (C) ELISA for FLAG-tagged cell-surface receptors shows dose-dependent loss of receptors from cell surface one h after spiradoline treatment. After a 1 μM dose of spiradoline, internalization is evident within 15 minutes. Data are expressed as a percentage of receptors detected on surface of untreated cells expressing Rog. Error bars represent SEM for three replicates.

Since cell-surface expression of a GPCR can be stabilized by the addition of antagonist [21,22], we examined the effect of the KOR antagonist norBNI on cell-surface expression of Rog-μA. Antagonist treatment nearly doubled the amount of Rog-μA detected in the membrane (Figure 4A; p < 0.005; F_1,6 _= 27.00). It also increased the cell-surface expression of Rog-μ, but to a lesser degree (p < 0.05, F_1,6 _= 11.54). In contrast, it had no effect on the cell-surface expression of Rog. The increase in membrane expression of Rog-μA after antagonist treatment was confirmed by confocal microscopy. Under basal conditions, little Rog-μA was seen in the plasma membrane, although the receptor was readily detected in other areas of the cell (Figure 4B). After overnight treatment with 10 μM norBNI, most of the Rog-μA was seen in the plasma membrane (Figure 4B). In contrast, untreated Rog receptors were primarily located in the plasma membrane (Figures 2B, 4B), and norBNI treatment had little effect on their localization (Figure 4B). These experiments suggest that Rog-μA may be constitutively downregulated and rapidly cycled in and out of the plasma membrane.

### Rog-A is more resistant to desensitization

In addition to regulation by internalization, a GPCR signal can be modulated by desensitization: uncoupling from the signaling effectors after continuous agonist stimulation. To explore desensitization directly, we briefly pretreated each RASSL with 1 nM spiradoline for 15 min, and examined inhibition of cAMP accumulation in response to a variety of doses of spiradoline. The low pretreatment dose had caused no receptor internalization detectable by ELISA-based assays. Pretreatment reduced the responsiveness of Rog receptors to spiradoline (Figure 5A, Table 1). The same maximal inhibition of cAMP accumulation was observed, but the dose response curve was shifted approximately 10-fold, with the EC50 for Rog shifting from 0.41 nM to 4.32 nM spiradoline after spiradoline pretreatment. Pretreatment of Rog-A with the same dose of spiradoline, however, did not significantly affect the response of the cells to subsequent treatment (Figure 5A, Table 1). The EC50 for spiradoline after 1 nM spiradoline pretreatment of Rog-A was 0.42 nM, compared to 0.41 nM for vehicle-treated cells. Spiradoline pretreatment shifted the EC50 of Rog-μ to 2.05 nM (Figure 5A, Table 1). Notably, the maximal response of pretreated Rog-μ-expressing cells was less than half that of untreated cells, indicating a decreased efficacy of Rog-μ signaling through the G_i _pathway. Spiradoline pretreatment strongly reduced the response of Rog-μA to further spiradoline treatment (Figure 5A, Table 1). In fact, it appears that the response to spiradoline in pretreated Rog-μA cells is so low that the dose range tested (up to 100 nM) does not yield a maximal inhibition of cAMP, and no sigmoidal dose-response curve can be fitted to these data. Therefore, we cannot calculate an accurate EC50 for desensitized Rog-μA receptors. However, assuming that the maximal response occurs at doses higher than 100 nM spiradoline, we can estimate that the EC50 would be at least 13.44 nM. This indicates that Rog-μ and Rog-μA receptors desensitize readily. For these receptors, the dose of spiradoline required to achieve the EC50 is significantly lower than the dose required to internalize the cell-surface receptors (Figures 4, 5). While only a fraction of the total surface receptor pool needs to be activated to activate G_i _maximally, a much larger fraction of the receptor population must be internalized before it can be accurately measured.

### AC superactivation is independent of receptor internalization

Chronic treatment of cells expressing G_i_-coupled receptors with agonist results in a compensatory increase in the activity of AC and, therefore, an increased accumulation of cAMP in response to the same dose of forskolin [3]. We examined the development of this AC superactivation in cell lines transiently expressing the RASSL variants. Forskolin (10 μM) stimulates twice as much cAMP in Rog-expressing cells treated with 10 nM spiradoline for 18 hours, compared to cells acutely treated with forskolin alone (Figure 5B; p < 0.005, F_1,10 _= 17.72). A similar degree of superactivation was seen in cells transfected with the wild-type KOR, indicating the same cellular response to prolonged G_i _signaling through both Rog and KOR. Overnight treatment of Rog-A-expressing cells with spiradoline, followed by stimulation with 10 μM forskolin, resulted in a slightly smaller increase in cAMP (Figure 5B; p < 0.05, F_1,10 _= 9.52). Notably, cells expressing Rog-μ and Rog-μA receptors showed no evidence of AC superactivation after 18 h of spiradoline pretreatment. These data show that receptors that desensitize and internalize more readily at the receptor level, such as Rog-μ and Rog-μA, do not induce compensations in an opposing signaling pathway.

Although little internalization of these RASSLs has been observed at these low doses of spiradoline, we wanted to ensure that the AC superactivation data could not be explained by differences in receptor internalization. Therefore, we performed an analysis of cell-surface receptor expression in parallel with the cAMP response experiment. Cells were plated and treated with 10 nM spiradoline for 18 hours exactly as described above. Then the cells were fixed and a cell-surface ELISA was performed. In general, 7–10% of the receptors were internalized by this treatment, but there were no significant differences between receptor types (Figure 5C). ANOVA showed a significant treatment effect (F_1,18 _= 8.22, p < 0.01), but no effect of receptor type (p > 0.99).

## Discussion

We have engineered a series of RASSLs that inhibit cAMP after acute activation by spiradoline with equal efficacy but differ dramatically in cellular location and responses to chronic drug treatment.

**Figure 3:**
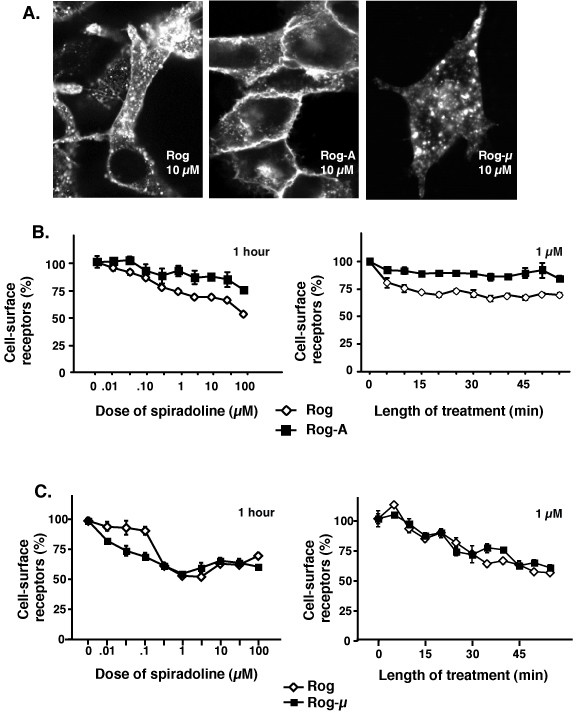
**Agonist-induced internalization is reduced in Rog-A, but not Rog-μ. **(A) Confocal micrographs showing localization of GFP-tagged Rog (left), Rog-A (center), or Rog-μ (right) stably expressed in HEK293 cells and treated with 10 μM spiradoline for 1 h before fixation. (B) A dose-response ELISA shows less internalization of Rog-A in response to 1 h of spiradoline at doses of 0.3–100 μM spiradoline. After treatment with 1 μM spiradoline, Rog-A showed less internalization up to 1 h after treatment. (C) A dose-response ELISA shows more internalization of Rog-μ in response to 1 h of low doses of spiradoline ranging from 0.01 to 0.1 μM. After treatment with 1 μM spiradoline, there was no difference in the time course of internalization between Rog and Rog-μ. ELISA data are expressed as a percentage of receptors detected on surface of untreated cells expressing the same receptor.

Mutation of phosphorylation sites on the C-terminus to alanines resulted in a receptor that was relatively resistant to internalization in the presence of moderate doses of agonist (Rog-A, Figure 3) and showed no significant desensitization after pretreatment with a low dose of agonist (Figure 5A). This is consistent with recent findings that mutation of a single serine to alanine is sufficient to block internalization and desensitization of the KOR, since this mutation removes a residue that is required for G protein receptor kinase (GRK2) phosphorylation [23]. While these studies highlight the importance of GRK phosphorylation of GPCRs in mediating receptor internalization and desensitization, it is notable that partial internalization of Rog-A was still detected in response to higher doses of spiradoline, indicating that the receptor can be internalized through different mechanisms. Reports of GPCR endocytosis in the absence of GRK phosphorylation [24-26] suggest that the removal of C-terminal phosphorylation sites may reduce the affinity of the receptor for proteins that mediate endocytosis without preventing the protein-protein interactions that are essential for internalization.

**Figure 4:**
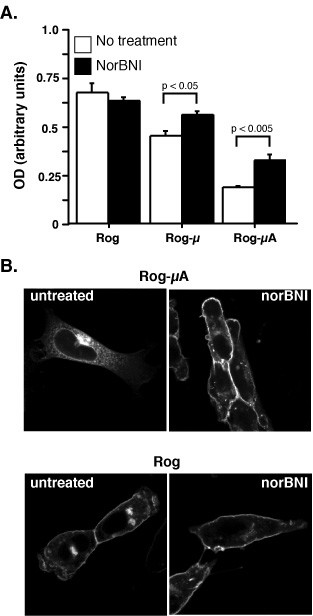
**Antagonist treatment increases cell-surface expression of Rog-μA. **(A) ELISA comparing cell-surface expression of receptors stably expressed in HEK293 cell lines, either untreated (white bars) or treated for 18 h with the antagonist norBNI (10 μM, dark bars). Error bars represent SEM for three replicates. NorBNI significantly increased cell-surface expression of both Rog-μ and Rog-μA. OD, optical density. (B) Confocal micrographs showing that the localization of GFP-tagged Rog-μA is primarily intracellular in untreated cells (left), but the receptor moves to the membrane after 18 hours of antagonist treatment (right). There is relatively little change in Rog after norBNI treatment relative to untreated cells.

**Figure 5:**
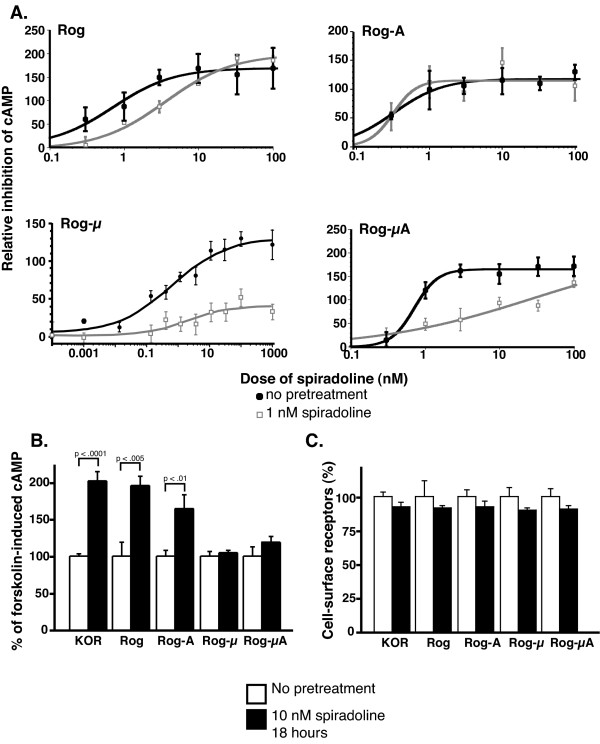
**Desensitization of cAMP inhibition and superactivation of AC after pretreatment with spiradoline **(A) Spiradoline (1 nM pretreatment) inhibited forskolin-induced cAMP formation in HEK293 cells transiently expressing Rog, Rog-A, Rog-μ, and Rog-μA. Data are expressed as inhibition of forskolin-induced cAMP. The baseline (0) represents maximum forskolin-induced cAMP accumulation in control cells. Pretreatment with 1 nM spiradoline for 10 min causes a shift in the dose-response curve for later spiradoline treatment in Rog and Rog-μA cells, but not Rog-A. Spiradoline pretreatment lowered the maximal response of Rog-μ to subsequent spiradoline treatment. (B) HEK293 cells transiently expressing receptors were treated 18 h with 10 nM spiradoline, and assayed for cAMP accumulation in response to a 15-min treatment with 10 μM forskolin. Spiradoline pretreatment significantly increased forskolin-induced cAMP in cells expressing KOR, Rog and Rog-A. Pretreatment of cells expressing Rog-μ and Rog-μA had not effect on response to forskolin. Data are expressed relative to the amount of cAMP accumulated after 10 μM forskolin treatment in cells pretreated with vehicle. Bars represent mean ± SEM for six replicates per condition. (C) HEK293 cells transiently expressing receptors were treated 18 h with 10 nM spiradoline, and assayed for loss of cell-surface expression by ELISA. Long-term spiradoline treatment significantly reduces cell-surface expression of all receptors, but the degree of internalization does not differ among different receptors. Bars represent mean ± SEM for three replicates per condition.

Rog-μ, the MOR/KOR chimeric mutant, was more sensitive to agonist-induced desensitization (Figure 5A) and internalization (Figure 3) than Rog. This is consistent with observations that the MOR internalizes and desensitizes more readily than the KOR. Surprisingly, mutation of C-terminal phosphorylation sites on the MOR/KOR chimera to form Rog-μA did not inhibit desensitization (Figure 5A). Rog-μA has low basal surface expression, although the GFP-tagged receptor can be seen throughout the cell (Figure 4B). It is unlikely that the extracellular mutations in Rog-μA are responsible for the unusual internalization pattern of this receptor. The extracellular mutations in Rog-μA are identical to the ones in Rog, which was shown to reach the cell surface and respond to agonist the same as GFP-tagged wild-type KOR (Figure 2). The intracellular pool of Rog-μA is not due to receptor misfolding or abnormal sorting, because some receptor was detected on the membrane (Figure 4A) and the receptor showed normal agonist-induced signaling (Figure 5A, Table 1). The observation that the addition of an antagonist can "rescue" the low cell-surface expression of Rog-μA (Figure 4A) further suggests that misfolding is not responsible for the decrease in cell surface expression.

There are several potential mechanisms for the increase in cell-surface expression after antagonist treatment. One possibility is that the antagonist, norBNI, acts as a molecular chaperone, entering the cell, binding to the receptor in intracellular compartments, and bringing it to the membrane. Ligands can act as pharmacological chaperones for the delta opioid receptor, facilitating receptor maturation and export from the endoplasmic reticulum [21]. However, there are no reports that the norBNI antagonist is cell-permeable. Another possibility is that norBNI acts as an inverse agonist, stabilizing cell-surface receptors in an "off" conformation, making them inaccessible to GRKs and arrestins, which usually interact only with active receptors. This would suggest that in the absence of norBNI, Rog-μA may be constitutively active. However, the receptor still signals robustly in response to spiradoline (Figure 5A), so it cannot be fully active in the absence of ligand. It is also possible that, under basal conditions, Rog-μA has a higher than normal affinity for GRK or arrestin, but not the G proteins. This would result in constitutive turnover – the receptor constantly cycling in and out of the membrane – in the absence of constitutive signaling. The idea that this receptor is especially sensitive to the desensitization and internalization machinery is borne out by the observation of extensive desensitization in response to pretreatment with a low dose of agonist (Figure 5A). It will be interesting to investigate the physiological consequences of this apparent constitutive internalization and rapid desensitization in animal models.

Although most of the C-terminal sequence of Rog-μA is derived from the MOR, the MOR does not exhibit either constitutive turnover or abnormally low membrane expression. There is some evidence for partial basal internalization of the similar Rog-μ receptor (Figure 4A), although Rog-μ appears to be expressed predominantly at the cell surface under basal conditions. Since Rog-μ and Rog-μA differ at only five amino acids, some of those five residues must be responsible for the increased turnover of Rog-μA. Although phosphorylation of T394 has been reported to be required for desensitization of the MOR [18], subsequent reports have shown that mutating T394 to alanine facilitates the internalization and resensitization of the receptor [27]. This suggests that phosphorylation of T394 may be a membrane retention signal and that the T394A mutation in Rog-μA is responsible for the constitutive endocytosis observed in this study. Mutagenesis of individual amino acids in this region may allow the identification of the specific residue(s) responsible for the constitutive internalization of Rog-μA.

The differences in AC superactivation between our RASSLs indicate another layer of complexity in GPCR signaling. Previous studies suggest an inverse correlation between the ability of an opioid receptor to undergo ligand-activated endocytosis and its ability to induce AC superactivation by chronic signaling [28]. The induction of superactivation by Rog is consistent with this idea. Rog-μ, which internalizes and desensitizes readily, failed to induce any AC superactivation after chronic activation (Figure 5B). Similarly, Rog-μA, which may undergo constitutive endocytosis, did not induce AC superactivation after chronic administration of spiradoline. However, Rog-A, which was predicted to have enhanced superactivation due to its resistance to endocytosis, had levels of superactivation comparable to those seen with Rog. It is possible that Rog induces maximal superactivation, and the cell cannot respond to Rog-A signaling with any additional superactivation. Our results indicate that internalization does not directly induce AC superactivation. The dose of spiradoline (10 nM over 18 hours) used to induce strong AC superactivation in these experiments causes only minimal internalization of all of the receptors (Figure 5C). Moreover, there is no significant difference in degree of internalization among the different receptor types, although they show profound differences in superactivation. This suggests that the cellular mechanisms underlying AC superactivation and receptor internalization are independent.

AC superactivation has been attributed to upregulation of AC proteins induced by Gβγ protein subunits interacting directly with GRK2/3 proteins [29,30]. Therefore, the same mutations that prolong Rog-A signaling and inhibit endocytosis may also prevent the receptor from interacting with GRK2/3 proteins and subsequently activating Gβγ-mediated signaling events. Alterations in desensitization characteristics are unlikely to alter AC superactivation because of the drastic differences in time course underlying these distinct phenomena. Receptor desensitization happens on a scale of minutes, while AC superactivation is the result of much longer term chronic receptor activation. Therefore, a decrease in G_i _signaling due to a more desensitized receptor is unlikely to have a significant effect on AC superactivation over the much longer time course used in these experiments. The additional possibility exists that altering C-terminal residues on the RASSL could increase the ability of receptors to couple to G_o_, resulting in perceived changes in AC superactivation [31]. However, if this were the case, one would expect to see a shift in the dose response curve for cAMP inhibition between Rog, Rog-A, Rog-μ and Rog-μA that is not observed in any of our experiments. Further studies with this engineered receptor system *in vivo *may clarify the complex relationship between ligand dependent endocytosis, interaction of a GPCR C-terminus with GRK2/3, desensitization, and superactivation of AC.

The ability of these RASSLs to induce different degrees of AC superactivation may have important physiological consequences *in vivo*. Interestingly, when a RASSL with a C-terminus corresponding to the wild-type human KOR was expressed at high levels in the hearts of transgenic mice, the mice developed a lethal cardiomyopathy [32]. One possible explanation is that basal signaling of the RASSL in mouse heart may increase G_s _signaling through AC superactivation. G_s _signaling has long been associated with heart failure, so AC superactivation may be responsible for the cardiomyopathy. Rog-μ and Rog-μA, RASSLs that do not induce superactivation, could be used to test this hypothesis and to study the consequences of AC superactivation in other tissues.

Internalized opioid receptors can be either degraded or recycled back to the membrane. The receptors that return to the membrane are stripped of arrestin, phosphates, and ligand, and are resensitized to ligand. Recent evidence demonstrates that the C-terminus is crucial for directing internalized receptors either into the degradative lysosomal pathway or back to the plasma membrane [27,33]. We expect that several of our engineered RASSLs should also differ in their post-endocytotic fate.

It is likely that the complex mechanisms governing GPCR endocytosis, recycling, desensitization and AC superactivation will be regulated differently in different cell types. The RASSLs described here exhibit similar properties in several different mammalian cell lines we tested (rat1a, CHO and HEK293), but their properties may change in specific cell types or under specific physiological conditions. One potentially fruitful avenue for future investigations would be to target different RASSLs to particular cell types *in vivo*. This would allow a thorough investigation of the interplay between receptor sequence and cell-type specific mechanisms of receptor regulation.

The development of a toolbox of engineered RASSLs that differ in internalization and desensitization raises several possibilities for future research and clinical investigations. Growing evidence points to a link between receptor dynamics and the potential for drugs to elicit tolerance or dependence, especially for opioid receptors [28,34]. Here, we present a system that allows the same drug to activate different receptors that have small, well-defined variations in sequence. The efficacy and potency of spiradoline is similar for Rog, Rog-A, Rog-μ and Rog-μA (Table 1); only the desensitization and internalization responses vary. Specifically, agonist-induced internalization is reduced in Rog-A and enhanced in Rog-μ, while Rog-μA shows agonist-independent internalization. Rog-A shows no desensitization after a brief spiradoline pretreatment, while the same treatment reduces the potency of spiradoline at Rog-μA and reduces efficacy at Rog-μ. Longer spiradoline pretreatment induces normal AC superactivation at Rog-A, but does not affect the AC response in Rog-μ or Rog-μA cells.

A family of engineered GPCRs that do not respond to endogenous ligands has enormous potential for selectively controlling G protein signaling in specific tissues without interfering with endogenous processes. Rog-A, a long-signaling RASSL, has several interesting implications for *in vivo *signal engineering. The basic RASSL, Ro2, shows rapid and extensive physiological desensitization [14], making it difficult to use in any therapeutic context where repeated activation of the receptor would be necessary. Rog-A, with its reduced desensitization, could allow continued physiological efficacy of repeated drug treatments. Comparison of the regulation and signaling of Rog, Rog-A, Rog-μ, Rog-μA, and future variants will contribute to the growing understanding of how GPCR signals are dynamically modulated. Study of these RASSLs *in vivo *will help solidify the elusive links between the receptor amino acid sequence, cell biology, and complex physiology.

## Methods

### Construction of mutant receptors

All receptors were based on the human kappa-opioid RASSL called Ro2 [12]. The GFP-tagged version of the RASSL has been named "Rog" for RASSL opioid with GFP tag. Rog was made by inserting the coding sequence for emerald GFP (Packard) at the N-terminus of the receptor, after a FLAG tag (DYKDDDDV) and the first eight amino acids of the RASSL. To create Rog-A, two serines and two threonines in the C-terminal region of the receptor were mutated to alanines (Figure 1). In the human kappa opioid receptor (KOR), the mutated residues correspond to S356A, T357A, S358A and T363A. The exact location of those residues in our RASSL construct, and the complete sequence of all RASSL variants can be found on our web site . For both Rog-μ and Rog-μA, the final 35 amino acids (345–380) of Rog were replaced by 47 C-terminal residues from the rat mu opioid receptor (MOR). Rog-μA contains the following additional modifications to the rat MOR C-terminus: T383A, E388Q, E391Q, E393Q and T394A. For each receptor, a schematic design and a C-terminal amino acid sequence alignment is shown in Figure 1. All constructs were sequenced to verify the mutations.

### Expression of RASSLs in mammalian cells

HEK293 cells were grown in culture to 60–80% confluence and then transfected using Lipofectamine Plus (Invitrogen, Carlsbad, CA). The RASSL construct contained a cytomegalovirus promoter to drive mammalian expression, and a neomycin-resistance gene to allow selection of stable cell lines. Experiments on transiently transfected cells were performed approximately 48 h after transfection. To create stable cell lines, transfected cells were selected with G418 (500 μg/ml, Invitrogen) for 10–14 days. Individual colonies showing green fluorescence were selected and grown under maintenance doses of G418 (250 μg/ml). Receptor expression was confirmed visually by fluorescence microscopy, and by an enzyme-linked immunoadsorbent assay (ELISA, see below).

### Cell-surface ELISA

Cell-surface expression of receptors was confirmed by an ELISA that detects only extracellular FLAG tag, which labels the N terminus of all RASSLs. This assay therefore quantifies only receptors that are in the membrane at the time of labeling, without providing detailed localization data about those receptors. Cells were plated at 100,000 cells/well on to 24-well plates coated with poly-d-lysine. Cultured cells were fixed in 4% paraformaldehyde for 10 min at 4°C, washed in phosphate-buffered saline (PBS), and then incubated in 1 μg/ml M1 anti-FLAG antibody (Sigma, St. Louis, MO) for 1 h at room temperature. They were washed again in PBS with 1 mM CaCl_2 _and incubated for 30 min at room temperature in secondary antibody (1:1000 goat anti-mouse conjugated with horseradish peroxidase, Biorad, Chicago, IL), then washed three times in PBS plus CaCl_2_. To develop the reaction, 0.25 ml of 2,2-azino-bis(3-ethylbenzthiazoline-6-sulfonic acid) liquid substrate (Sigma) was added to each well. After 15–60 min, 200 μl from each well was transferred to a 96-well plate and the optical density was read at 410 nm. To quantify agonist-induced internalization, cells were treated with various doses of spiradoline in medium for 10–120 min. The medium was removed and the cells were fixed and processed as described above. Vehicle treated cells on each plate were used to calculate a "maximum" cell-surface expression for that plate. All other treatment conditions on that plate were then normalized to this maximum to determine "percent internalization." Each experiment included 3–6 replicates per condition and was repeated at least 3 times. After values for cell-surface expression of each receptor were calculated and normalized, receptor expression was compared using two-way ANOVA (StatView v. 5.0, SAS Institute, Cary, NC). For dose response studies, receptor and dose were independent factors. For time course studies, receptor and length of treatment were independent factors.

### cAMP accumulation assay

The degree of cAMP inhibition in spiradoline-treated HEK-293 cells transiently expressing RASSL variants was measured with the CatchPoint cAMP ELISA kit (Molecular Devices, Sunnyvale, CA). Cells were plated at 5 × 10^4^/well into 96-well plates coated with poly-d-lysine. The next day, cells were rinsed in Krebs-Ringer bicarbonate buffer with glucose (KRBG, Sigma). Cells were then incubated in pre-stimulation buffer containing a phosphodiesterase inhibitor (0.75 mM 3-isobutyl-1-methylxanthine in KRBG buffer) for 10 min at room temperature to inhibit cAMP degradation. cAMP production was stimulated by the addition of 50 μM forskolin to all cells. At the same time, various doses of spiradoline in PBS were added to cells to establish a dose-response curve. After a 15-min drug treatment at 37°C, the cells were lysed and cAMP accumulation was assayed according to the CatchPoint protocol. Inhibition of cAMP by spiradoline was determined by comparison to cells treated with forskolin alone. For each experiment, 3–6 wells per condition were averaged, and EC50 and percent inhibition values for each receptor were determined by fitting curves for each independent experiment using SOFTmax PRO v. 4.0.1 (Molecular Devices). Data for 3–8 independent experiments were averaged to determine the EC50 and maximum cAMP inhibition for each receptor and condition. For desensitization assays, a pretreatment dose (1 nM) of spiradoline diluted in sterile PBS was added to the cells, which were then incubated for 10 min at 37°C. The cells were rinsed 4 times in KRBG and then stimulated and assayed for cAMP as described above. AC superactivation was determined by measuring forskolin-stimulated (10 μM) cAMP after treatment with either 10 nM spiradoline or vehicle for 18 h. AC superactivation data were expressed as a percent increase in forskolin-stimulated cAMP relative to vehicle-pretreated cells expressing the same receptor. Conditions and receptors were compared using a two-way ANOVA with receptor and treatment condition as independent factors.

### Confocal microscopy

HEK293 cells stably expressing receptor constructs were plated at a density of 500,000 cells/ml onto glass Labtek II chamber slides (Fisher Scientific, Pittsburgh, PA) coated with poly-d-lysine. The following day, the cells were treated with agonist (spiradoline or dynorphin A 1–13) for typically one hour, or antagonist (NorBNI) overnight and briefly washed in PBS. The PBS was removed and replaced with 1 ml of cold 4% paraformaldehyde in PBS. The cells were fixed at room temperature for 10 min, washed with PBS, and then mounted in Vectashield (Vector Laboratories, Burlingame, CA) under cover slips. For confocal imaging on a Bio-Rad MRC 600 microscope, typical images were taken with a 40–60× oil immersion objective lens, subject to 5× Kalman filtering. The microscope operator was blind to both cell line and treatment condition.

### Fluorometric imaging plate reader (FLIPR) assay

All receptors tested were transiently transfected using Lipofectamine into CHO cells in conjunction with the chimeric G protein G_qi5 _[20] in a 5:1 molar ratio of DNA. G_qi5 _is a chimeric G protein alpha subunit that has the G_αq _wild-type sequence except for the C-terminal 5 residues, which were changed to the corresponding G_αi _sequence. This allows G_i_-coupled receptors to signal through the G_q _pathway, resulting in a signal that can be detected using calcium-sensitive reagents. The following day, the cells were plated in a 96-well plate (50,000 cells per well) and allowed to grow for 24 h before being incubated with the calcium-sensitive dye fura-3 for 1 h. The assay was performed as described [12] using a range of dilutions of either spiradoline or dynorphin A 1–13 peptide (Sigma). All experiments were performed in triplicate.

## Authors' contributions

KSL and MDL created the constructs and cell lines used here, designed and conducted the experiments; analyzed the data; and drafted the manuscript. HHE maintained cell lines and participated in making constructs, internalization assays and confocal microscopy. BRC conceived of the study and participated in its design and interpretation. All authors read and approved the final manuscript.

## Figures and Tables

**Table 1:** EC50 and Emax values for inhibition of cAMP accumulation by spiradoline

	**Rog**	**Rog-A**	**Rog-μ**	**Rog-μA**
	**EC50 (nM spiradoline)**			

Acute activation	0.41 ± 0.08	0.41 ± 0.15	0.42 ± 0.23	0.50 ± 0.22

1 nM spiradoline pretreatment	4.32 ± 1.96	0.42 ± 0.21	2.05 ± 0.03	> 13.44 ± 5.41

	**% Inhibition**			

Acute activation	64.4 ± 4.8	56.1 ± 5.1	58.0 ± 0.9	64.1 ± 4.8

1 nM spiradoline pretreatment	55.7 ± 3.7	53.3 ± 0.9	43.6 ± 1.5	51.5 ± 6.0

Values are mean ± SEM for 3–8 experiments for each condition.

## References

[B1] Ferguson SSG (2001). Evolving concepts in G protein-coupled receptor endocytosis: The role in receptor desensitization and signaling. Pharmacol Rev.

[B2] Paing MM, Stutts AB, Kohout TA, Lefkowitz RJ, Trejo J (2002). β-arrestins regulate protease-activated receptor-1 desensitization but not internalization or down-regulation. J Biol Chem.

[B3] Avidor-Reiss T, Nevo I, Levy R, Pfeuffer T, Vogel Z (1996). Chronic opioid treatment induces adenylyl cyclase V superactivation. J Biol Chem.

[B4] Kieffer BL, Evans CJ (2002). Opioid tolerance – In search of the holy grail. Cell.

[B5] Borgland SL, Connor M, Osborne PB, Furness JB, Christie MJ (2003). Opioid agonists have different efficacy profiles for G protein activation, rapid desensitization, and endocytosis of mu-opioid receptors. J Biol Chem.

[B6] Alvarez VA, Arttamangkul S, Dang V, Salem A, Whistler JL, Von Zastrow M, Grandy DK, Williams JT (2002). mu-Opioid receptors: Ligand-dependent activation of potassium conductance, desensitization, and internalization. J Neurosci.

[B7] Zimmer M, Palmer A, Kohler J, Klein R (2003). EphB-ephrinB bi-directional endocytosis terminates adhesion allowing contact mediated repulsion. Nat Cell Biol.

[B8] Haglund K, Sigismund S, Polo S, Szymkiewicz I, Di Fiore PP, Dikic I (2003). Multiple monoubiquitination of RTKs is sufficient for their endocytosis and degradation. Nat Cell Biol.

[B9] Nong Y, Huang YQ, Ju W, Kalia LV, Ahmadian G, Wang YT, Salter MW (2003). Glycine binding primes NMDA receptor internalization. Nature.

[B10] Braithwaite SP, Xia H, Malenka RC (2002). Differential roles for NSF and GRIP/ABP in AMPA receptor cycling. Proc Natl Acad Sci USA.

[B11] St John PA, Gordon H (2001). Agonists cause endocytosis of nicotinic acetylcholine receptors on cultured myotubes. J Neurobiol.

[B12] Coward P, Wada HG, Falk MS, Chan SDH, Meng F, Akil H, Conklin BR (1998). Controlling signaling with a specifically designed G_i_-coupled receptor. Proc Natl Acad Sci USA.

[B13] Scearce-Levie K, Coward P, Redfern CH, Conklin BR (2001). Engineering receptors activated solely by synthetic ligands (RASSLs). Trends Pharmacol Sci.

[B14] Redfern CH, Coward P, Degtyarev MY, Lee EK, Kwa AT, Hennighausen L, Bujard H, Fishman GI, Conklin BR (1999). Conditional expression and signaling of a specifically designed G_i_-coupled receptor in transgenic mice. Nat Biotechnol.

[B15] Zhao GQ, Zhang Y, Hoon MA, Chandrashekar J, Erlenbach I, Ryba NJ, Zuker CS (2003). The receptors for mammalian sweet and umami taste. Cell.

[B16] Capeyrou R, Riond J, Corbani M, Lepage J-F, Bertin B, Emorine LJ (1997). Agonist-induced signaling and trafficking of the μ-opioid receptor: Role of serine and threonine residues in the third cytoplasmic loop and C-terminal domain. FEBS Lett.

[B17] El Kouhen R, Burd AL, Erickson-Herbrandson LJ, Chang C-Y, Law P-Y, Loh HH (2001). Phosphorylation of Ser^363^, Thr^370^, and Ser^375 ^residues within the carboxyl tail differentially regulates μ-opioid receptor internalization. J Biol Chem.

[B18] Pak Y, O'Dowd BF, George SR (1997). Agonist-induced desensitization of the μ opioid receptor is determined by threonine 394 preceded by acidic amino acids in the COOH-terminal tail. J Biol Chem.

[B19] Schulz R, Wehmeyer A, Schulz K (2002). Visualizing preference of G protein-coupled receptor kinase 3 for the process of κ-opioid receptor sequestration. Mol Pharmacol.

[B20] Conklin BR, Farfel Z, Lustig KD, Julius D, Bourne HR (1993). Substitution of three amino acids switches receptor specificity of G_q_α to that of G_i_α. Nature.

[B21] Petäjä-Repo UE, Hogue M, Bhalla S, Laperrière A, Morello J-P, Bouvier M (2002). Ligands act as pharmacological chaperones and increase the efficiency of δ opioid receptor maturation. EMBO J.

[B22] Morello J-P, Salahpour A, Laperrière A, Bernier V, Arthus M-F, Lonergan M, Petäjä-Repo U, Angers S, Morin D, Bichet DG, Bouvier M (2000). Pharmacological chaperones rescue cell-surface expression and function of misfolded V2 vasopressin receptor mutants. J Clin Invest.

[B23] McLaughlin JP, Xu M, Mackie K, Chavkin C (2003). Phosphorylation of a carboxy-terminal serine within the kappa opioid receptor produces desensitization and internalization. J Biol Chem.

[B24] Bouvier M, Hausdorff WP, De Blasi D, O'Dowd BF, Kobilka BK, Caron MG, Lefkowitz RJ (1988). Removal of phosphorylation sites from the β_2_-adrenergic receptor delays onset of agonist-promoted desensitization. Nature.

[B25] Ferguson SSG, Ménard L, Barak LS, Koch WJ, Colapietro A-M, Caron MG (1995). Role of phosphorylation in agonist-promoted β_2_-adrenergic receptor sequestration. J Biol Chem.

[B26] Qiu Y, Law PY, Loh HH (2003). μ-opioid receptor desensitization: Role of receptor phosphorylation, internalization and representation. J Biol Chem.

[B27] Wolf R, Koch T, Schulz S, Klutzny M, Schröder H, Raulf E, Bühling F, Höllt V (1999). Replacement of threonine 394 by alanine facilitates internalization and resensitization of the rat μ opiod receptor. Mol Pharmacol.

[B28] Finn AK, Whistler JL (2001). Endocytosis of the mu opioid receptor reduces tolerance and a cellular hallmark of opiate withdrawal. Neuron.

[B29] Chakrabarti S, Oppermann M, Gintzler AR (2001). Chronic morphine induces the concomitant phosphorylation and altered association of multiple signaling proteins: A novel mechanism for modulating cell signaling. Proc Natl Acad Sci USA.

[B30] Pitcher JA, Freedman NJ, Lefkowitz RJ (1998). G protein-coupled receptor kinases. Annu Rev Biochem.

[B31] Watts VJ (2002). Molecular mechanisms for heterologous sensitization of adenylate cyclase. J Pharmacol Exp Ther.

[B32] Redfern CH, Degtyarev MY, Kwa AT, Salomonis N, Cotte N, Nanevicz T, Fidelman N, Desai K, Vranizan K, Lee EK, Coward P, Shah N, Warrington JA, Fishman GI, Bernstein D, Baker AJ, Conklin BR (2000). Conditional expression of a Gi-coupled receptor causes ventricular conduction delay and a lethal cardiomyopathy. Proc Natl Acad Sci USA.

[B33] Whistler JL, Enquist J, Marley A, Fong J, Gladher F, Tsuruda P, Murray SR, von Zastrow M (2002). Modulation of postendocytic sorting of G protein-coupled receptors. Science.

[B34] Bohn LM, Gainetdinov RR, Lin FT, Lefkowitz RJ, Caron MG (2000). Mu-opioid receptor desensitization by beta-arrestin-2 determines morphine tolerance but not dependence. Nature.

